# Medical faculty members’ perception of smartphones as an educational tool

**DOI:** 10.1186/s12909-019-1697-5

**Published:** 2019-07-17

**Authors:** Oqab Jabali, Munther Saeedi, Ghada Shbeitah, Abed Alkarim Ayyoub

**Affiliations:** 1 Language Center, Faculty of Humanities, An-Najah National University, Nablus, Palestine; 2 Faculty of Educational Sciences and Teachers’ Training, An-Najah National University, Nablus, Palestine

**Keywords:** Attitudes, Medical education, Medical faculty members, Smartphones, Perception, Teaching tool

## Abstract

**Background:**

The rapid adoption of modern technology has changed many aspects of our life and communication; it has the power to influence and change the way we teach, learn and practice different types of professions mainly teaching and health care providing. Smartphone applications are increasingly becoming popular and widespread. Generally, these applications are likely to play a significant role in supporting education, in general, and medical education, in particular. This study aims at investigating how medical faculty members are using smartphones in medical education and practice, and how they perceive them as an educational tool at university level.

**Methods:**

The researchers have distributed an online questionnaire - including three parts: a demographic part with five variables; a 15-item part of various applications of the smartphones; and a 14-item part measuring attitudes towards using these smartphones - among medical faculty members at two Palestinian universities.

**Setting and participants:**

Medical faculty members working at two Palestinian universities. Data have been collected from 30 participants out of 72 representing a response rate of 41.6%.

**Results:**

The average skills score with smartphones usage is (3.18) which tells that faculty members use smartphones to support their teaching practices. In general, faculty members are positive towards smartphones as a prospective teaching tool since the average attitude towards using smartphones is (3.60). The study results show no significant differences among faculty members based on the five demographic variables, i.e. university, title, department affiliation, gender, and years of experience.

**Conclusion:**

It seems that the majority of faculty members believe that smartphones would be a significant instrument as well as addition to their teaching practices.

**Electronic supplementary material:**

The online version of this article (10.1186/s12909-019-1697-5) contains supplementary material, which is available to authorized users.

## Background

With the development of new technologies (e.g., smartphones) and the multiplicity of their functions and widespread use compared to other devices, smartphones have recently started to come to the forefront. Many people contend that they have become part and parcel of everyone’s life regardless of age, sex or economic level of their users. Undoubtedly, having a smartphone means coping up with the internet age; the effective use of smartphones is an important indicator of computer knowledge or literacy, ([12]; [24]; and [18]). Due to the incredible versatility and internet capabilities of smartphones, educators are beginning to stress and highlight their advantages and take serious steps to minimize their disadvantages.

Smartphones provide educators and learners with opportunities to collect, assess, and process knowledge and information inside and outside classrooms; they promote learning in a real-world context, collaboration and communication that is adaptable to individual needs and diverse learners’ levels [26]. Using smartphones changes teaching methods [30], learner roles, and even the place in which education takes place [10].

Recent advances in information and communication technology has led to integration into university teaching and learning processes ([21, 22]). Traditional pedagogy includes a teacher-centered approach with the passive learners that are unable to determine their learning style [1].

In contrast, in a learner-centered classroom, students are actively involved and they have greater input into what they learn, how they learn it, and when they learn it [4]. Learner-centered teaching means that students take responsibility of their own learning and are directly involved in the learning process with a focus on how students learn instead of how teachers teach [28, 29]. Consequently, different countries have recently commenced introducing technology and smartphones, in particular, in their schools and teaching centers. They contend that technological changes are a positive challenge to schools, and a means of bringing teaching into the smart technology age. Therefore, use of smartphones may facilitate flexible learner-centered approach that is void of the restrictions of time and place and develops learners’ leadership skills, teamwork, creativity, communication, collaboration, critical thinking and problem solving [3, 19].

A large number of universities have started to incorporate smartphones in the teaching/leaning process. [8] reported that integration of smartphones into teaching and learning at three United States universities improved student collaboration and integration while utilizing social media and Web 2.0 tools. Similarly, [5] suggested the likelihood of using smartphones as a platform for collaborative educational experiences. Cochrane [2] suggested that smartphones could be useful when incorporated in the teaching/learning process in the sense that they help learner get to relevant knowledge and improve their collaboration among each other and with their instructors as well.

From a pure medical perspective, a number of studies were conducted to test the various applications of smartphone in the teaching/learning process. Australian dental students used their smartphones as a learning tool to look at the timetable and course announcements, surfed the internet for learning material, and took notes and pictures of their work [18]. Canadian medical teachers and learners employed their smartphones to enhance not only learning but also the way respondents take care of their patients [27]. Another research was carried out at University of Birmingham, UK found that 37% of medical students showed understanding and positive perceptions towards their smartphone usage and application and that they used their devices to enhance learning [17].

Various types of technology can be used to socialize, learn, share, and create material via open collaboration. Despite the many barriers to using smartphones in education, technologies are very likely to deepen learning and enhance dental students’ involvement in the teaching/learning process [13]. Integrating new media tools in medical curricula is also possible. In the United States, study findings showed that incorporating such tools in faculty coursework boosted and increased not only learning but also teamwork, let alone promoting certain skills including problem solving and networking [7].

Despite the many benefits of technology, smartphones may have side-effects. On the one hand, they are likely to distract students in classes [23]. On the other hand, they have detrimental effects on users’ health let it be students or otherwise. The Electromagnetic Radiation of smartphones is harmful and leads to negative side effects [6, 9, 14].

Most of the literature relates to students’ or learners’ perceptions about their smartphones as a tool of study. However, very few studies have been carried out to attest the instructors’ perceptions towards using their smartphones as a teaching tool. This present study was mainly intended to fill in this gap. Therefore, the purpose of this article was to (1) investigate medical university educators use of smartphones as a teaching tool; and (2) the educators’ perceptions towards using smartphones in teaching. Put simply, the researchers aimed to answer the following two questions: (1) How do faculty members use smartphones to support their teaching? and (2) What are faculty members’ perceptions towards using smartphones as a teaching tool?

## Methods

Ethical approval was obtained from An-Najah National University vice president of academic affairs, the dean of the faculty of medicine, the dean of scientific research department, as well as the research ethics committee. A descriptive online questionnaire was built to explore the ways medicine university instructors perceive their smartphones as a possible teaching tool in two Palestinian universities. As the researchers were interested in examining relevant information efficiently and logically, smartphone use as a teaching tool was conceived comprehensively to include any possible application or benefit that might soothe, improve, and facilitate instruction at university level. The survey is divided into three sections (See Additional file 1). Part one introduces the independent variables of the study; it relates to demography (social and demographic features of the study sample including the university, title, department affiliation, gender and years of experience). Parts two and three represent the dependent variables. Part two consists of 15 items that represent the various applications of smartphones (including giving instructions, communication, accessing information, sharing information, etc.) as a teaching tool. A five-point Likert Scale, with Very Often (5), Often (4), Occasionally (3), Rarely (2), and Never (1), has been used to measure the frequency of the 15 items. The last part consists of 14 items which relate to lecturers’ perceptions towards the use of the smartphones as educational devices. A five-point Likert Scale, with Strongly Agree (5), Agree (4), Uncertain (3), Disagree (2), Strongly Disagree (1), has been used to measure the 14 agreement items.

**Table 1:** Smartphones general scores reported as mean and SD

Item	Mean	Standard Deviation
I send emails to my students to discuss subject content and attach course outline and other important information.	3.27	1.46
I access and download textual materials, audio and video clips for my class directly from my smartphone.	2.90	1.47
I contact my students for important information.	4.37	.72
I use text messages to send notifications (class cancellations, change of lecture venue, change in time of lectures and other administrative duties).	3.50	1.41
I encourage students to submit their assignments online from their smartphones.	2.87	1.46
I have course materials such as slides, lecture notes and practice quizzes available on my smartphone.	2.90	1.60
I read news, books and articles online directly from my smartphone in order to gather more information on topics treated in class.	3.87	1.25
I use online dictionaries on my smartphone to get definitions/meanings related to topics in my class.	3.87	1.33
I use Bluetooth from my smartphone to share materials with my students.	1.80	.92
I download materials onto my smartphone to store up-to-date information for my class.	2.93	1.55
I access textbooks that are available via the Internet or ebook readers.	3.73	1.11
I use my smartphone as a timer and an alarm in classes and exams.	3.23	1.38
I do library /literature searches and reserve some book for future borrowings.	3.07	1.39
I allow their students to snap photos of the chalkboard or whiteboard as class wraps up in case they couldn’t finish taking their notes fast enough.	3.47	1.57
I use my smart phone to check attendance in the classroom.	1.97	1.33
General Question: How do lecturers use smartphones to support their teaching?	3.18	.84

The first draft of the online questionnaire had undergone various types of content validity by (6) experts in the field of questionnaire development. They deleted some items and included others to maintain content validity of the questionnaire particularly face validity. Simple associations were conducted to ensure convergent validity. The results showed that the items are highly associated as the lowest value (item 14) of question 1 is (0.573) and it is higher than the highest value of items related to question 2 (item 8) which is 0.549). To ensure the reliability of the questionnaire, Cronbach’s Alpha was calculated. Cronbach’s Alpha coefficient was 0.88 for the first question items; it was 0.92 for the second question items and for the total items the coefficient alpha was 0.95. The three alpha values were higher than 0.7; this shows the questionnaire is reliable.

### Sample

There are four universities (An-Najah National University in Nablus, Arab American University in Jenin, Al-Quds University in Jerusalem, and Hebron University in Hebron) in the West Bank, Palestine that teach medicine. The sample was chosen from the first two universities. Al-quds University was excluded for logistic considerations; it is very difficult for the researchers to get to the university because of Israeli occupation. Hebron University recently started the medicine program; it began 2018/2019. One university in Gaza teaches medicine; it was excluded for logistic considerations.

The population of this study included university instructors who teach at the faculty of medicine (92 faculty members); (60) work for An-Najah National University and (32) for at the Arab American University. However, few faculty members answered the survey (*n* = 30): 27 faculty members from An-Najah University and three only from the Arab American University. Data collection was carried out during the second semester of the academic year 2018/2019.

### Analysis

Data was normally distributed and was analyzed using means and percentages. Chi-square tests were used to calculate the associations between demographic elements and scores; student T-test was used when there are two variables and ANOVA was used when demographic elements compared are more than two, while linear regression was used to assess associations between demographics and perceptions. The data was analyzed using SPSS version 21. Absolute values are used with percentages to indicate unanswered questions. Associations were tested at 95% significance level (*P* < .05).

## Results

### Demographic characteristics

In total, 41.6% (30/72) of faculty members from the two universities answered the online questionnaire. Of the 72 faculty members, 60 (83.6%) work at An-Najah National University; only 27/60 responded to the questionnaire. 12 (16.4%) faculty members work for the Arab American University in Jenin; only three answered the questionnaire. These faculty members have various titles; Professors represent (6.7%), Associate Professors (6.7%), Assistant Professors (46.7%), Instructors (20%), and Lecturers (20%). With respect to members’ department affiliation, half the respondents teach at the department of medicine and more than a quarter work at the department of bio-medical sciences. The least number of respondents (3.3%) come from the department/faculty of nursing. With respect to gender, half of the respondents are males and the rest are females. And with respect to experiences, the respondents are divided equally; (33.3%) has been working for less than 5 years, (33.3%) has been working for five to 10 years, and (33.3%) has been working for more than 10 years.

### Assessment of smartphone use

**Fig. 1:**
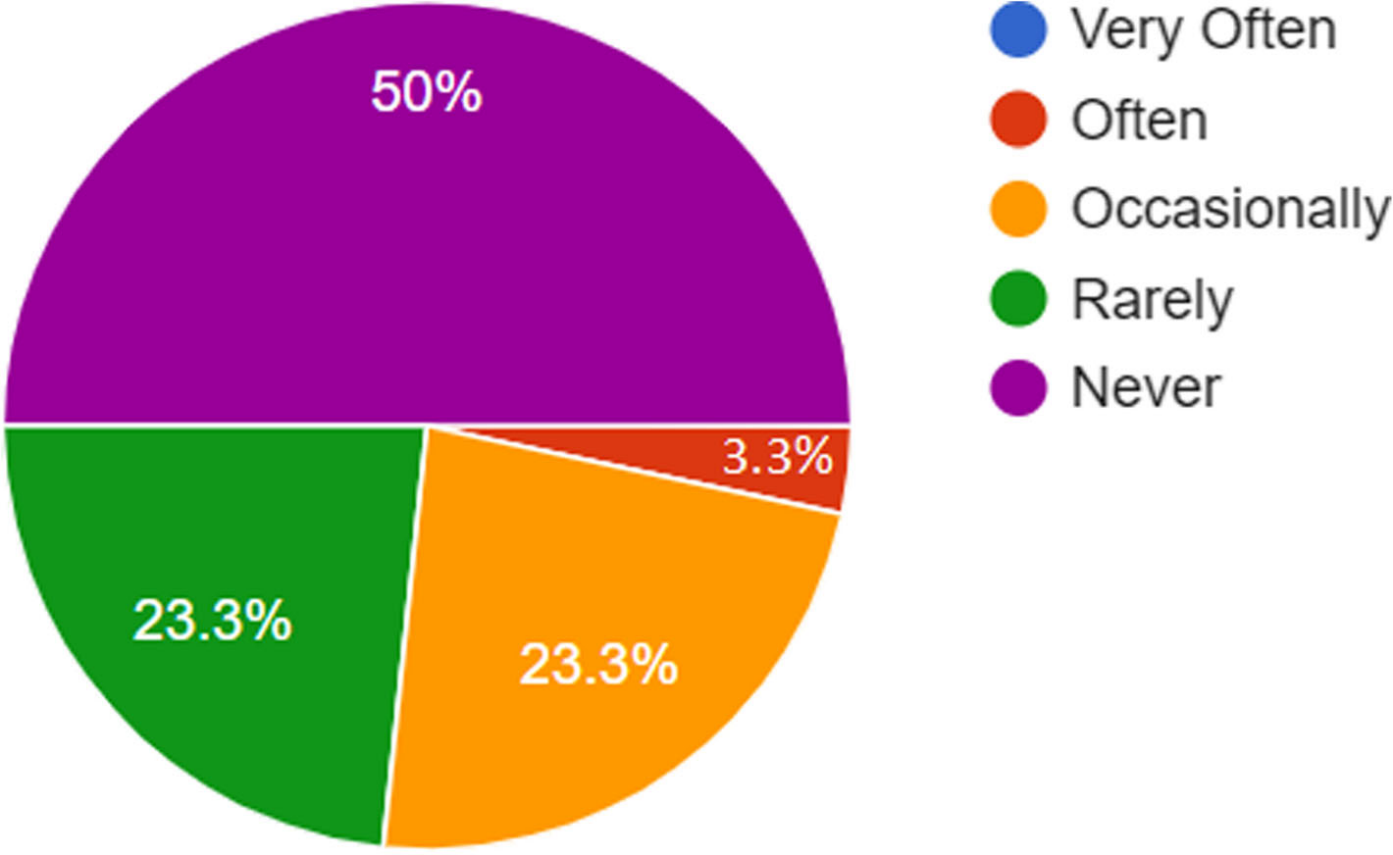
Representations of faculty members’ frequency of contacting students with smartphones

The average skills score with smart phones usage was (3.18). As Table 1 below shows, almost all faculty members used their smartphones to contact their students for important information (mean = 4.37, SD = 0.72). This means that most responses were close to either very often or often, see Fig. 1.

**Table 2:** Statistical results related to uses of smartphones based on the five variables

Demographic Feature	Mean	Std. Deviation	*P*
University			
An-Najah National University	3.106	.8467	.038
Arab American University in Jenin	3.866	.3711	
Gender			
Male	3.097	.8398	.591
Female	3.266	.8615	
Title			
Professor	4.33	.19	.606
Associate professor	3.93	.38	
Assistant professor	3.04	.86	
Lecturer	2.92	.64	
Instructor	3.44	.68	
Department Affiliation			
Medicine	3.34	.75	.890
Bio-medical	3.11	.91	
Nursing	2.87	1.00	
Dentistry	3.70	.33	
Years of experience			
Less than 5 years	3.29	.59	.277
From 5 to 10 years	2.90	1.11	
More than 10 years	3.36	.74

**Fig. 2:**
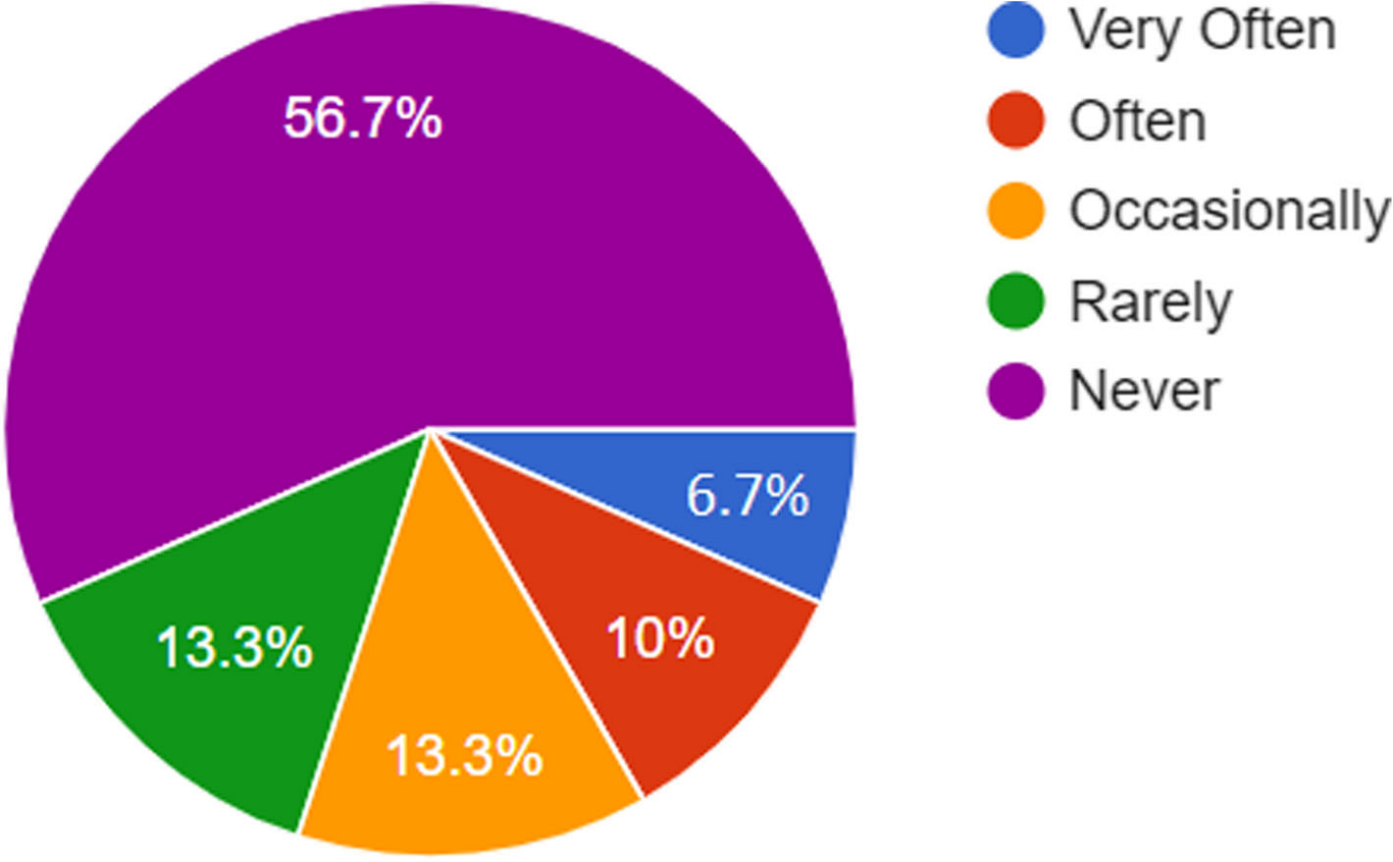
Representations of faculty members’ frequency of checking attendance with smartphones

Three-quarters of the respondents did not share materials with students using the Bluetooth in their smartphones (mean = 1.8) and did not use the smart phone to check attendance in classes (mean = 1.97). Figure 2 shows that (56.7%) of the respondents never did this via their smartphones. It is worth mentioning that attendance at these two university is compulsory.

There were no significant differences among faculty members attributed to all demographic features, i.e. the university the respondent teaches in, the department affiliation, the title, the years of experiences and the gender as shown in Table 2 below.

There were no statistical differences between faculty members attributed to their title (*P* = .606). There were also no statistical differences between faculty members attributed to their department affiliation (*P* = .890). It is worth mentioning that one faculty member teaches at the department of pharmacy; therefore, he has not been considered in the analysis. As far as the experience is concerned, one-third of the respondents have been teaching for less than 5 years, one-third works between five to 10 years, and the rest have more than 10 years; nevertheless, no significant differences were found as (*P* = .277).

### Assessment of perceptions towards smartphone use

The average attitude towards using smart phones was positive (mean = 3.60); faculty members at the two universities were in favor of using their smart phones for different purposes in the teaching process, Table 3.

**Table 3:** General scores of faculty members’ perceptions towards smartphones reported as mean and SD

Item	Mean	Standard Deviation
Smartphones are useful as a supplementary to teaching.	3.73	1.17
Smartphones improve access to my courses and learning material.	3.60	1.38
Smartphones help me organize my work better.	3.77	1.01
Smartphones enhance easier access to information anywhere and anytime.	4.20	1.00
Text messaging via smartphones is useful as an instructional tool in class.	3.20	1.30
Shooting videos of lectures allows students who miss class or may not have caught something the first time.	3.57	1.10
Smartphones can increase in class participation and elsewhere collaboration between students.	2.90	1.16
Smartphones increase communication between the lecturer and the student.	3.37	1.30
Smartphones can help students be more prepared for class by easily accessing information before class.	3.27	1.14
Smartphones provide students with the opportunity to work at their own pace.	3.27	1.14
Smartphones allow students to get access to up-to-date information through the Web and social media.	4.00	1.02
Smartphones can green up the classroom by converting as many class materials to digital as possible.	3.37	1.10
Smartphones can encourage students to store everything on their smartphones, Tablets, computers, or other device.	4.13	1.01
Smartphone features allow users to learn grammar, spelling, pronunciation, and other essential literacy skills.	4.03	.85
Total question: What are faculty members’ perceptions towards using smartphones as a teaching tool?	3.60	.90

**Fig. 3:**
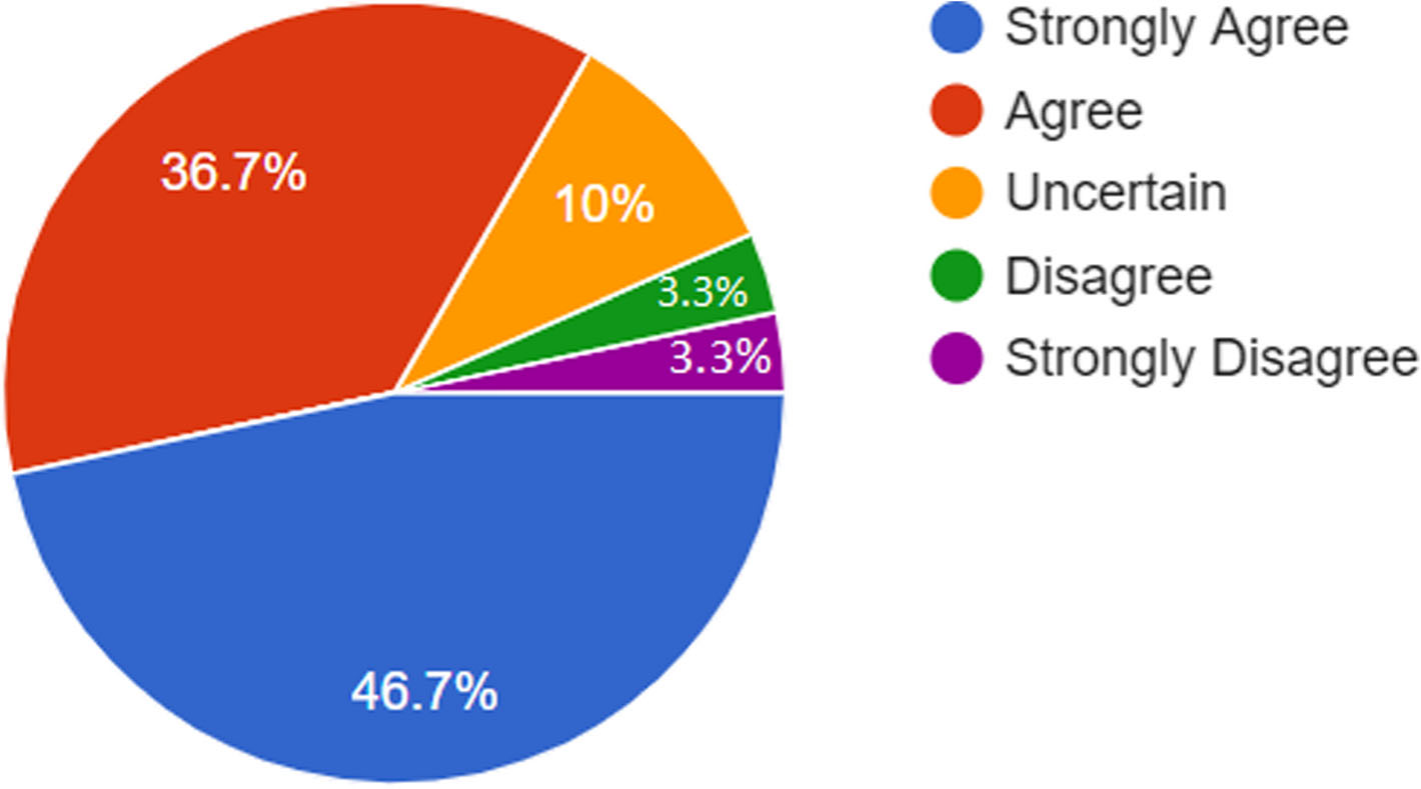
Representations of faculty members’ perceptions towards enhancing easier access to information

**Fig. 4:**
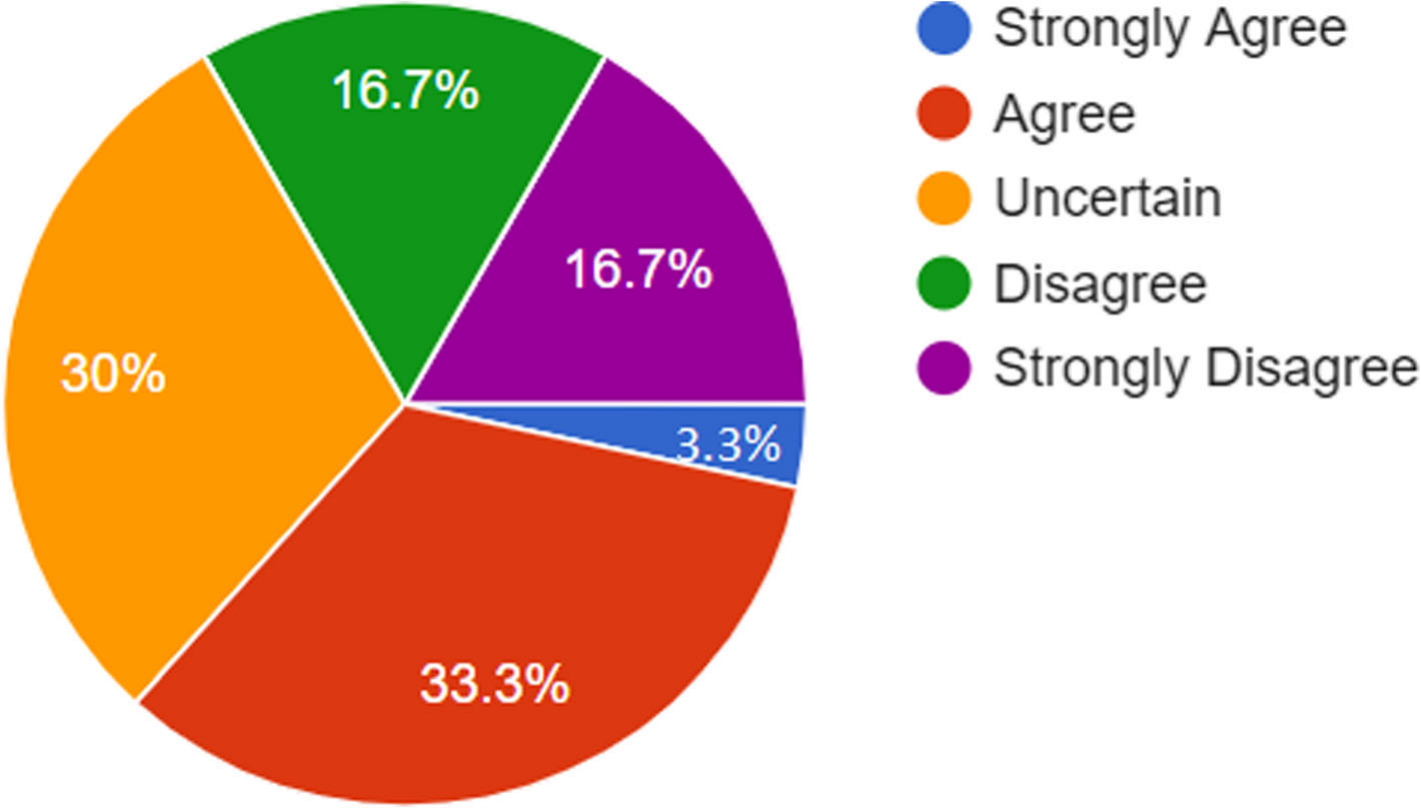
Representations of faculty members’ perceptions towards increasing in-class participation and collaboration

As can be noticed from Table 4 above, the majority of faculty members believed that Smartphones enhance easier access to information anywhere and anytime (mean = 4.20 and SD = 1.00). Figure 3 shows that the majority of respondents (90%) indicated positive perceptions towards smartphones in the sense that they encourage students store everything they need for study. However, one-third of the respondents had unclear perceptions towards smartphones as a means of increasing in-class participation and collaboration among students and (33.3%) of faculty members was uncertain, see Fig. 4.

**Table 4:** Statistical results related to faculty members’ perceptions towards smartphones based on all demographic variables

Demographic Feature	Mean	Std. Deviation	*P*
University			
An-Najah National University	3.5608	.94006	.119
Arab American University in Jenin	3.9524	.25085	
Gender			
Male	3.5571	.99386	.800
Female	3.6429	.82949	
Title			
Professor	4.25	.05	.067
Associate professor	4.39	.45	
Assistant professor	3.48	.73	
Lecturer	3.31	1.11	
Instructor	3.69	1.22	
Department Affiliation			
Medicine	3.77	.74	.890
Bio-medical	3.50	.60	
Nursing	3.64	.1.52	
Dentistry	3.82	.15	
Years of experience			
Less than 5 years	3.73	.52	.277
From 5 to 10 years	3.23	1.26	
More than 10 years	3.84	.72

The statistical analysis of faculty members’ perceptions towards using smart phones showed no significant differences among faculty members attributed to all demographic features, i.e. the university the respondent teaches in, the department affiliation, the title, the years of experiences and the gender. Study results showed no differences between them based on the university they work for and/or their gender.

With respect to title, department affiliation and years of experience, the One-way ANOVA tests showed that there were no statistical differences between faculty members attributed to their title (*P* = .067). There were also no statistical differences between faculty members attributed to their department affiliation (*P* = .890). It is worth mentioning that one faculty member teaches at the department of pharmacy; therefore, he has not been considered in the analysis. As far as the experience is concerned, no significant differences were found as (*P* = .277) as shown in Table 4 below.

## Discussion

The study was aimed to investigate medical university educators’ use of smartphones as a teaching tool and their perceptions towards using these smartphones in teaching. The results of the current study revealed that most faculty members have smart phones which they use in the teaching process at medicine faculties in the two Palestinian universities. They perceive the smarts as teaching tools that help them carry out their mission smoothly and accurately.

Smartphones were reported by a large portion of the study sample as a tool that enables them to contact their students, read news, books and articles online from smartphones in order to gather more information on topics treated in classes. The faculty members use online dictionaries on their smartphone to get definitions/meanings related to topics in class. They also access textbooks that are available via the Internet or ebook readers to enrich their resources. These results are consistent with other previous studies (e.g., [16, 20]). The study results showed no significant differences among respondents based on all the variables investigated.

Using smartphones in classes and lectures is likely to be a productive activity that improves student collaboration and integration and critical thing. Such findings were also seen in many previous studies such as ([2, 3, 5, 8, 18, 19]).

The study findings demonstrated that faculty members had positive perceptions towards their smart phones as seen in [17] and that there were no significant differences among faculty members based on the five demographic variables. The majority of faculty members agreed that smartphones enhance easier access to information anywhere and anytime; they also allow students to get access to up-to-date information through the Web and the social media; the same results were seen in many previous studies such as [11, 15, 25]. Smartphones can also deepen and boost learning and promote problem solving and networking skills. Similar results were shown by [7, 13].

Although smart phones have many positive attributes, they can be of no value for some including faculty members to the extent that few of them completely ban using them in classes as they may distract students; therefore, they do not use their smarts themselves. A good portion of faculty members did not use their smartphones to share materials with their students in classes as they think smartphones may distract students’ attention. Similar findings were seen in [23].

## Conclusion

The results of the current study corroborated that faculty members used smart phones in the teaching process despite the fact that this technology has not been formally allowed by the university administrations. Most of the respondents perceived their smartphones as an effective teaching tools to some extent. This might be an opportunity for more teaching staff in other faculties and universities to use smartphones to enhance students’ learning needs without the constraints of time and location. In light of the results of this study, it appears feasible to develop learning activities involving smartphones. It might be advisable to design learning material that not only allows access through LCDs or computers but also through smartphones.

In the realm of information technology advances, what is plausible in medicine may not be so in other disciplines. This study had several limitations; on top of them is the sample size. This might be an opportunity for more teaching staff in other faculties and universities in Palestine and elsewhere to use smartphones to enhance students’ learning needs without the constraints of time and location. Another limitation was the study tool. The questionnaire was answered by faculty members who might have not reported what they actually do with their smartphones. Thus, further research could use other methods to ensure accurate results. This study had not explored the opinion of administrative staff of educational institutions. Therefore, the researchers recommend conducting a study which investigates administration staffs’ perceptions of this technology as a teaching tool. The researchers had not compared the attitudes of faculty members with students towards incorporating this technology in the teaching/learning process. Finally, a prospective study might investigate the various applications of smartphones by medical practitioners as faculty members and doctors in clinics and/or hospitals.

## Additional file

Additional file 1: Supplementary survey. (DOCX 19 kb)

## Data Availability

Not applicable.

## Abbreviation

LCD: Light Crestal Display
