# Supplementary material for: Medical faculty members’ perception of smartphones as an educational tool
Source: BMC Med Educ. 2019 Jul 17;19:264. doi: 10.1186/s12909-019-1697-5 (PMC6637609; doi:10.1186/s12909-019-1697-5)
Supplement: Supplementary file 1 — Supplementary survey. (DOCX 19 kb) [file 12909_2019_1697_MOESM1_ESM.docx]

Additional file 1

Note: This questionnaire was sent electronically to faculty members in a form of a link via emails. Respondents were supposed to click the link and the questionnaire is displayed. Here is the link:

https://docs.google.com/forms/d/e/1FAIpQLSe6JGDN7peaetwkiYLzh-2aYRB3LheNzdAefcsfFV_m7ylYSA/viewform?usp=sf_link

Medical Faculty Members’ Perception of Smartphones as an Educational Tool

**Oqab Jabali**

Language Center, Faculty of Humanities, An-Najah

National University, Nablus Palestine

Email: [oqab.jabali@najah,edu](mailto:oqab.jabali@najah,edu)

**Munther Saeedi**

Language Center, Faculty of Humanities, An-Najah

National University, Nablus Palestine

Email: munther.saeedi@najah.edu

**Ghada Shbeitah**

Language Center, Faculty of Humanities, An-Najah

National University, Nablus Palestine

Email: ghada.odwan@najah.edu

**Abed Alkarim Ayyoub**

Faculty of Educational Sciences and Teachers’ Training, An-Najah

National University, Nablus, Palestine

Email: ayyoub@najah.edu

Please fill this questionnaire to help us to identify instructors’ uses and attitude towards smartphones in the teaching process. The aim of this questionnaire is to obtain information to incorporate new trends of teaching and instruction at university level. All data provided will remain confidential. By completing this questionnaire, you are indicating your willingness to participate. Your participation is greatly appreciated.

**Section A**

**Demographics and Social Characteristics**

**1- Title**: Instructor □ Lecturer □ Assistant professor □

Associate Professor □ Professor □

**2. University Name**: An-Najah □ American University in Jenin □

**3. Department Affiliation**: Pharmacy □ Medicine □ Dentistry □ Nursing □ Bio-medical Sciences □

**4. Gender**: Male □ Female □

**5. Years of experience**: Less than 5 years □ Five to 10 years □ More than 10 years□

**Section B**

**How lecturers use smartphones to support their teaching**

| **No.** | **Item** | **Very Often** | **Often** | **Occasionally** | **Rarely** | **Never** |
| --- | --- | --- | --- | --- | --- | --- |
| 1 | I send emails to my students to discuss subject content and attach course outline and other important information. |  |  |  |  |  |
| 2 | I access and download textual materials, audio and video clips for my class directly from my smartphone. |  |  |  |  |  |
| 3 | I contact my students for important information. |  |  |  |  |  |
| 4 | I use text messages to send notifications (class cancellations, change of lecture venue, change in time of lectures and other administrative duties). |  |  |  |  |  |
| 5 | I encourage students to submit their assignments online from their smartphones. |  |  |  |  |  |
| 6 | I have course materials such as slides, lecture notes and practice quizzes available on my smartphone. |  |  |  |  |  |
| 7 | I read news, books and articles online directly from my smartphone in order to gather more information on topics treated in class. |  |  |  |  |  |
| 8 | I use online dictionaries on my smartphone to get definitions/meanings related to topics in my class. |  |  |  |  |  |
| 9 | I use Bluetooth from my smartphone to share materials with my students. |  |  |  |  |  |
| 10 | I download materials onto my smartphone to store up-to-date information for my class. |  |  |  |  |  |
| 11 | I access textbooks that are available via the Internet or ebook readers. |  |  |  |  |  |
| 12 | I use my smartphone as a timer and an alarm in classes and exams. |  |  |  |  |  |
| 13 | I do library /literature searches and reserve some book for future borrowings. |  |  |  |  |  |
| 14 | I allow their students to snap photos of the chalkboard or whiteboard as class wraps up in case they couldn’t finish taking their notes fast enough. |  |  |  |  |  |
| 15 | I use my smart phone to check attendance in the classroom. |  |  |  |  |  |

**Section C**

**Lecturers’ attitudes towards using smartphones as a teaching tool**

| **Item No.** | **Item** | **Strongly**  **Agree** | **Agree** | **Uncertain** | **Disagree** | **Strongly**  **Disagree** |
| --- | --- | --- | --- | --- | --- | --- |
| 1 | Smartphones are useful as a supplementary to teaching. |  |  |  |  |  |
| 2 | Smartphones improve access to my courses and learning material. |  |  |  |  |  |
| 3 | Smartphones help me organize my work better. |  |  |  |  |  |
| 4 | Smartphones enhance easier access to information anywhere and anytime. |  |  |  |  |  |
| 5 | Text messaging via smartphones is useful as an instructional tool in class. |  |  |  |  |  |
| 6 | Shooting videos of lectures allows students who miss class or may not have caught something the first time. |  |  |  |  |  |
| 7 | Smartphones can increase in class participation and elsewhere collaboration between students. |  |  |  |  |  |
| 8 | Smartphones increase communication between the lecturer and the student. |  |  |  |  |  |
| 9 | Smartphones can help students be more prepared for class by easily accessing information before class. |  |  |  |  |  |
| 10 | Smartphones provide students with the opportunity to work at their own pace. |  |  |  |  |  |
| 11 | Smartphones allow students to get access to up-to-date information through the Web and social media. |  |  |  |  |  |
| 12 | Smartphones can green up the classroom by converting as many class materials to digital as possible. |  |  |  |  |  |
| 13 | Smartphones can encourage students to store everything on their smartphones, Tablets, computers, or other device. |  |  |  |  |  |
| 14 | Smartphone features allow users to learn grammar, spelling, pronunciation, and other essential literacy skills. |  |  |  |  |  |
